# Rural and Urban Differences in Air Quality, 2008–2012, and Community Drinking Water Quality, 2010–2015 — United States

**DOI:** 10.15585/mmwr.ss6613a1

**Published:** 2017-06-23

**Authors:** Heather Strosnider, Caitlin Kennedy, Michele Monti, Fuyuen Yip

**Affiliations:** 1 Environmental Health Tracking Branch, Division of Environmental Hazards and Health Effects, National Center for Environmental Health, CDC

## Abstract

**Problem/Condition:**

The places in which persons live, work, and play can contribute to the development of adverse health outcomes. Understanding the differences in risk factors in various environments can help to explain differences in the occurrence of these outcomes and can be used to develop public health programs, interventions, and policies. Efforts to characterize urban and rural differences have largely focused on social and demographic characteristics. A paucity of national standardized environmental data has hindered efforts to characterize differences in the physical aspects of urban and rural areas, such as air and water quality.

**Reporting Period:**

2008–2012 for air quality and 2010–2015 for water quality.

**Description of System:**

Since 2002, CDC’s National Environmental Public Health Tracking Program has collaborated with federal, state, and local partners to gather standardized environmental data by creating national data standards, collecting available data, and disseminating data to be used in developing public health actions. The National Environmental Public Health Tracking Network (i.e., the tracking network) collects data provided by national, state, and local partners and includes 21 health outcomes, exposures, and environmental hazards. To assess environmental factors that affect health, CDC analyzed three air-quality measures from the tracking network for all counties in the contiguous United States during 2008–2012 and one water-quality measure for 26 states during 2010–2015. The three air-quality measures include 1) total number of days with fine particulate matter (PM_2.5_) levels greater than the U.S. Environmental Protection Agency’s (EPA’s) National Ambient Air Quality Standards (NAAQS) for 24-hour average PM_2.5_ (PM_2.5_ days); 2) mean annual average ambient concentrations of PM_2.5_ in micrograms per cubic meter (mean PM_2.5_); and 3) total number of days with maximum 8-hour average ozone concentrations greater than the NAAQS (ozone days). The water-quality measure compared the annual mean concentration for a community water system (CWS) to the maximum contaminant level (MCL) defined by EPA for 10 contaminants: arsenic, atrazine, di(2-ethylhexyl) phthalate (DEHP), haloacetic acids (HAA5), nitrate, perchloroethene (PCE), radium, trichloroethene (TCE), total trihalomethanes (TTHM), and uranium. Findings are presented by urban-rural classification scheme: four metropolitan (large central metropolitan, large fringe metropolitan, medium metropolitan, and small metropolitan) and two nonmetropolitan (micropolitan and noncore) categories. Regression modeling was used to determine whether differences in the measures by urban-rural categories were statistically significant.

**Results:**

Patterns for all three air-quality measures suggest that air quality improves as areas become more rural (or less urban). The mean total number of ozone days decreased from 47.54 days in large central metropolitan counties to 3.81 days in noncore counties, whereas the mean total number of PM_2.5_ days decreased from 11.21 in large central metropolitan counties to 0.95 in noncore counties. The mean average annual PM_2.5_ concentration decreased from 11.15 *μ*g/m^3^ in large central metropolitan counties to 8.87 *μ*g/m^3^ in noncore counties. Patterns for the water-quality measure suggest that water quality improves as areas become more urban (or less rural). Overall, 7% of CWSs reported at least one annual mean concentration greater than the MCL for all 10 contaminants combined. The percentage increased from 5.4% in large central metropolitan counties to 10% in noncore counties, a difference that was significant, adjusting for U.S. region, CWS size, water source, and potential spatial correlation. Similar results were found for two disinfection by-products, HAA5 and TTHM. Arsenic was the only other contaminant with a significant result. Medium metropolitan counties had 3.1% of CWSs reporting at least one annual mean greater than the MCL, compared with 2.4% in large central counties.

**Interpretation:**

Noncore (rural) counties experienced fewer unhealthy air-quality days than large central metropolitan counties, likely because of fewer air pollution sources in the noncore counties. All categories of counties had a mean annual average PM_2.5_ concentration lower than the EPA standard. Among all CWSs analyzed, the number reporting one or more annual mean contaminant concentrations greater the MCL was small. The water-quality measure suggests that water quality worsens as counties become more rural, in regards to all contaminants combined and for the two disinfection by-products individually. Although significant differences were found for the water-quality measure, the odds ratios were very small, making it difficult to determine whether these differences have a meaningful effect on public health. These differences might be a result of variations in water treatment practices in rural versus urban counties.

**Public Health Action:**

Understanding the differences between rural and urban areas in air and water quality can help public health departments to identify, monitor, and prioritize potential environmental public health concerns and opportunities for action. These findings suggest a continued need to develop more geographically targeted, evidence-based interventions to prevent morbidity and mortality associated with poor air and water quality.

## Introduction

The physical and social aspects of the places in which persons live, work, and play that contribute to the development of disease are called social determinants of health (*1*). U.S. counties vary widely in terms of urbanization, with 6% of the population living in the most rural counties or county equivalents (i.e., <10,000 persons) and 31% living in the most urban counties (i.e., large central metropolitan counties of ≥1 million persons (*2*). A 2001 CDC report found significant differences in health among residents of counties with different urbanization levels, with the highest rates of death in the most rural and the most urban counties (*3*). The report also found significant differences in health behaviors, health care access, and risk factors, with the poorest outcomes in the most rural counties. Recent studies have found similar results, including a 2014 update to a CDC report by the Rural Health Reform Policy Center (*4*–*10*). In addition, evidence also indicates that national improvements in health such as an overall decrease in coronary heart disease have not equally affected highly rural areas (*6*). The associations between urbanization and health were frequently, but not always, found to be similar across categories defined by race/ethnicity, education, and poverty (*4*–*7*,*10*). In some cases, the difference between urban and rural areas was more substantial in poor areas compared with affluent areas (*5*,*10*). Studies also found geography or U.S. region could affect the association between urbanization and health (*5*,*7*,*9*). These studies suggest a complex relationship among urbanization, health, and social and demographic aspects of a particular environment, or place.

Although fewer studies have evaluated the associations of health with the physical aspects of a place, such as air or water quality, evidence suggests that such differences might correlate with differences in health (*11*–*13*). A place-based approach assesses the health needs in a population in relation to the unique interaction of contextual, structural, environmental, and ecological features of the geography. Efforts to characterize physical differences have been hindered by lack of nationally standardized environmental data (*14*). Environmental data often are collected for regulatory purposes and might be missing key elements (e.g., temporal or spatial data) that would facilitate a comprehensive environmental public health assessment. Since 2002, the National Environmental Public Health Tracking Program (i.e., the tracking program) at CDC has been working with federal, state, and local partners to address the lack of data by collecting and standardizing environmental data for analysis and dissemination on the National Environmental Public Health Tracking Network (i.e., the tracking network). The analysis in this report uses nationally standardized air- and water-quality data to evaluate how these environmental hazards vary across a spectrum of urban to rural counties. Understanding these differences using a place-based approach can help to identify, mitigate, and prevent the environmental exposures that contribute to chronic disease.

### Air

Particulate matter and ozone are two well-characterized air pollutants that can affect health and are monitored and regulated by the U.S. Environmental Protection Agency (EPA) (*15*,*16*). Particulate matter (solid or liquid particles suspended in the air) include smoke, fumes, soot, and combustion by-products, as well as natural particles (e.g., windblown dust, pollen, and sea salt). The transport and effect of particulate matter, both in the atmosphere and in the human respiratory tract, are governed principally by particulate size, shape, and density. Particulate matter is characterized by size as coarse, fine, or ultrafine. Particles with an aerodynamic diameter <2.5 *μ*m (PM_2.5_) are categorized as fine particulate matter. Ozone is a gas that occurs naturally in the stratosphere, approximately 10–30 miles above the Earth, and protects the Earth from the ultraviolet rays of the sun. Ozone also exists at ground level and is the primary component of smog. At ground level, ozone is created when specific pollutants react in the presence of sunlight. In urban areas, vehicular and industrial emissions are chief contributors to ozone production. Ground-level ozone adversely affects health and damages the environment.

The association between PM_2.5_ concentrations and acute and chronic adverse health outcomes includes premature death, lung cancer, exacerbation of respiratory and cardiovascular disease, and increased risk for cardiovascular morbidity (e.g., myocardial infarction and arrhythmia) (*16*). Populations most susceptible to these outcomes include older adults, children, and persons with heart and lung disease. National Ambient Air Quality Standards (NAAQS) were established by the Clean Air Act Amendments of 1970, which required EPA to set air-quality standards for specific pollutants such as PM_2.5_ and ozone to protect the health of the general public and of populations most at risk for pollutant-related adverse health outcomes.

Short-term exposures to ozone have been associated with an increase in deaths and in cardiovascular- and respiratory-related hospitalizations (*15*). Ozone exposure can result in lung and throat irritation, lung inflammation, wheezing, and difficulty breathing. Exposure to ozone can exacerbate bronchitis, emphysema, and asthma. Populations at risk for ozone-related health effects are those who typically spend long periods outdoors (e.g., persons with outdoor occupations and athletes) and sensitive groups, including infants and children, older adults, and persons with respiratory or cardiovascular disease.

### Water

Approximately 90% of persons in the United States get their drinking water from a public water system (PWS) (*17*). These systems are publically or privately owned and provide drinking water to at least 15 service connections or serve an average of at least 25 persons for at least 60 days a year. As required by the Safe Drinking Water Act, EPA sets regulatory limits known as maximum contaminant levels (MCLs) for approximately 90 contaminants in water provided by PWS. Drinking water protection programs at the state and national levels play a critical role in ensuring high-quality drinking water and in protecting public health. The tracking network has data for several contaminants that can be found in drinking water provided by community water systems (CWSs), which are PWSs that serve water to the same population year-round. The 10 contaminants available from the tracking network were selected because they were identified as priority contaminants by a workgroup with representatives from state and local health departments and environmental departments, CDC, and EPA. These contaminants include arsenic, atrazine, di(2-ethylhexyl) phthalate (DEHP), haloacetic acids (HAA5), nitrate, perchloroethene (PCE), radium, trichloroethene (TCE), total trihalomethanes (TTHM), and uranium. These contaminants are associated with a range of acute and chronic adverse effects (e.g., gastrointestinal illness, reproductive disorders, and cancer) (*18*). However, the risk for developing a specific disease depends on many factors, including the properties of the specific contaminant, the amount of contaminant to which a person is exposed, exposure pathways such as drinking or showering, and individual factors such as body size, age, preexisting health conditions, and health behaviors.

## Methods

The tracking network collects data provided by national, state, and local partners and includes 21 health outcomes, exposures, and environmental hazards. To assess environmental factors that affect health, CDC analyzed air- and water-quality data from the tracking network for various years. Findings are presented by urban-rural classification scheme: four metropolitan categories (large central metropolitan, large fringe metropolitan, medium metropolitan, and small metropolitan) and two nonmetropolitan categories (micropolitan and noncore). Regression modeling was used to determine whether differences in the measures by urban-rural categories were statistically significant.

The 2008–2012 tracking network air-quality data that were analyzed included three measures calculated using both monitoring and modeled air data from EPA and included all counties in the contiguous United States. The three measures include 1) total number of days with PM_2.5_ levels greater than NAAQS for 24-hour average PM_2.5_ (PM_2.5_ days); 2) mean annual average ambient concentrations of PM_2.5_ in micrograms per cubic meter (mean PM_2.5_); and 3) total number of days with maximum 8-hour average ozone concentrations greater than the NAAQS (ozone days). These measures were calculated using EPA air monitoring data supplemented with downscaler-modeled data for days and counties without monitoring data (*19*). Daily data were used to calculate the three annual measures of air quality, thus providing complete data for all counties in the contiguous United States during 2008–2012.

The 2010–2015 tracking network data that were analyzed for water quality were from the Safe Drinking Water Information System, provided by tracking program grantees or other state health departments for 28,350 CWSs in 26 states. The single measure used compared the annual mean concentration for a CWS to the MCL defined by EPA for each of 10 contaminants: arsenic, atrazine, DEHP, HAA5, nitrate, PCE, radium, TCE, TTHM, and uranium. The annual mean for each contaminant was calculated using all samples for a CWS in a year, including sample results below the limit of detection, which were set to half the detection limit. In some years, no samples were taken for a specific contaminant in a specific CWS because of the monitoring schedule set by the regulatory standards; therefore, no mean was calculated. The principle county served was identified for each CWS.

Air- and water-quality measures were linked by county to an urban-rural classification scheme developed by CDC (*2*), which classifies counties (or county-equivalent entities) based on the 2010 Office of Management and Budget delineation of metropolitan statistical areas (MSAs) and micropolitan statistical areas, population size of the MSA, and location of principal city within the MSA for the large counties (*20*). This scale has four categories of metropolitan counties and two categories of nonmetropolitan counties, for a total of six urbanization categories for counties. The metropolitan categories are large metropolitan (MSA population ≥1 million), medium metropolitan (MSA population 250,000–999,999) and small metropolitan (MSA population <250,000). Large metropolitan counties are subdivided into large central metropolitan counties and large fringe metropolitan counties based on the location and size of the MSA principal city. The nonmetropolitan categories are micropolitan (counties in a micropolitan statistical area defined as urban clusters with a population of 2,500–49,999) and noncore (counties not in micropolitan statistical area). Although urbanization decreases along a continuum from large central metropolitan counties to noncore counties, noncore counties were considered rural in this report. 

### Statistical Analysis

The mean and standard error across all counties by urbanization category were calculated for each air- and water-quality measure. In addition, regression modeling with a dummy variable for urbanization category with large central metropolitan counties as the reference category was used to evaluate differences between the most urban counties and increasingly less urban counties. To adjust for possible spatial correlation, a fixed effect for state was included in all models. Air- and water-quality measures were modeled differently depending on the characteristics of the data. Measures for the total number of PM_2.5_ days and the total number of ozone days were evaluated under the assumption that these measures were sampled from a Poisson distribution; therefore, rate ratios (RRs) were calculated using Poisson regression. The mean PM_2.5_ concentration was evaluated using a rate of change calculated by a linear model under the assumption that these data were sampled from a normal distribution. The probability a CWS reported at least one annual mean greater than the MCL was evaluated using a generalized estimating equation (GEE) logistic regression approach and an independent working correlation structure. A GEE model was used to account for the reporting of more than one annual mean during 2010–2015 from the same CWS in the data set. Odds ratios (ORs) were calculated and 95% confidence intervals (CIs) were estimated with GEE-based robust standard errors. Additional variables for U.S. region, CWS size, and water source were included in the model because they were identified as potential confounders (*21*). The CWS size variable included five categories based on population served, ranging from very small (<501 persons) to very large (>100,000 persons). The water source for each CWS was either surface water, groundwater, groundwater under direct influence of surface water, or unknown. Water treatment practices can vary by CWS size and by type of water source, and the occurrence of a contaminant in source water can vary by region and by type of water source. For all air- and water-quality measures, 95% CIs were calculated for the respective estimates (rate of change, RR, or OR), and differences between rural and urban areas were considered statistically significant if the CIs did not overlap. Caution should be used when using this conservative approach as an alternative for statistical testing because, although infrequent, differences might not be detected. 

## Results

### Air

During 2008–2012, the mean total number of ozone days ranged from 47.54 days in large central metropolitan counties to 3.81 days in noncore counties (Table 1). The total number of PM_2.5_ days during 2008–2012 ranged from a mean of 11.21 days in large central metropolitan counties to 0.95 days in noncore counties. The average annual PM_2.5_ concentration during 2008–2012 ranged from 11.15 *μ*g/m^3^ in large central metropolitan counties to 8.87 *μ*g/m^3^ in noncore counties. All three measures of air quality indicated that air quality decreased as counties became more urban. Large fringe metropolitan counties experienced a greater decrease than medium and small metropolitan counties in the total number of days greater than the PM_2.5_ standard compared with large central metropolitan counties. The regression analyses indicate that the decreases in air quality in all three measures for large central metropolitan counties compared with other less urban categories of counties are significant based on the 95% CIs (Table 1). For the total number of ozone days, the RR decreased from 0.62 for large fringe metropolitan counties to 0.17 for noncore counties. For the total number of PM_2.5_ days, the RR decreased from 0.54 for large fringe metropolitan counties to 0.23 for noncore counties. Large fringe metropolitan counties were associated with a -1.18 difference in average annual PM_2.5_ concentration, whereas noncore counties were associated with a -2.29 difference compared with large central metropolitan counties.

**TABLE 1:** Air­-quality measures in urban and rural counties — United States, 2008–2012

County urban-rural category* No. (%)	Air-quality measure^†^					
	Ozone days^§^		PM_2.5_ days^¶^		Average PM_2.5_ concentration (*µ*g/m^3^)**	
	Mean (SE)^††^	Estimate^§§^ (95% CI)	Mean (SE)^††^	Estimate^§§^ (95% CI)	Mean (SE)^††^	Estimate^§§^ (95% CI)
Large central metropolitan 68 (100)	47.54 (1.12)	Reference	11.21 (0.25)	Reference	11.15 (0.03)	Reference
Large fringe metropolitan 368 (100)	20.67 (0.09)	**0.62 (0.59 to 0.65)**	2.82 (0.01)	**0.54 (0.49 to 0.59)**	10.51 (0.004)	**-1.18 (-1.4 to -0.97)**
Medium metropolitan 370 (99)	13.89 (0.10)	**0.45 (0.43 to 0.47)**	4.93 (0.05)	**0.76 (0.7 to 0.83)**	10.17 (0.004)	**-1.33 (-1.55 to -1.12)**
Small metropolitan 355 (99)	7.95 (0.04)	**0.32 (0.3 to 0.33)**	3.55 (0.03)	**0.62 (0.56 to 0.68)**	9.85 (0.004)	**-1.63 (-1.85 to -1.41)**
Micropolitan 637 (99)	5.83 (0.01)	**0.23 (0.22 to 0.24)**	1.78 (0.01)	**0.35 (0.32 to 0.39)**	9.47 (0.003)	**-1.92 (-2.14 to -1.71)**
Noncore 1,311 (98)	3.81 (0.005)	**0.17 (0.16 to 0.18)**	0.95 (0.002)	**0.23 (0.21 to 0.25)**	8.87 (0.001)	**-2.29 (-2.49 to -2.08)**

**Abbreviations:** CI = confidence interval; EPA = U.S. Environmental Protection Agency; MSA = metropolitan statistical area; NAAQS = National Ambient Air Quality Standards; PM_2.5_ = particulate matter ≤2.5 microns in diameter (fine particulate matter); PPM = parts per million; SE = standard error.

* Number represents the number of counties in the data by urban-rural classification. Percent represents the percentage of U.S. counties in that urban-rural classification in the data. The metropolitan categories are large metropolitan (MSA population ≥1 million), medium metropolitan (MSA population 250,000–999,999) and small metropolitan (MSA population <250,000). Large metropolitan counties are subdivided into large central metropolitan counties and large fringe metropolitan counties based on the location and size of the MSA principal city. The nonmetropolitan categories are micropolitan (counties in a micropolitan statistical area defined as urban clusters with a population of 2,500–49,999) and noncore (counties not in a micropolitan statistical area) (**Source:** Ingram DD, Franco SJ. 2013 NCHS urban-rural classification scheme for counties. Vital Health Stat 2 2014;166:1–73).

^†^ Data for all three measures were obtained from the National Environmental Public Health Tracking Network and include 2008–2012 combined.

^§^ Total number of days with maximum 8-hour average ozone concentrations greater than EPA’s NAAQS of 0.070 ppm.

^¶^ Total number days with 24-hour average PM_2.5_ levels greater than EPA’s NAAQS of 35 *µ*g/m^3^.

** Annual average ambient PM_2.5_ concentration (*µ*g/m^3^).

^††^ Mean and mean SE of the air-quality measure for all counties in that urban-rural classification.

^§§^ Effect estimates for the total number of PM_2.5_ days and the total number of ozone days are odds ratios calculated with Poisson regression. Effect estimates for average PM_2.5_ concentration were calculated with linear regression. Estimates in bold are significant (based on the 95% CIs).

### Water

During 2010–2015, the data included 401,652 annual means for 10 contaminants reported by 28,350 CWSs representing 47% (n = 1,498) of all U.S. counties and 43% (n = 589) for all noncore U.S. counties to 58% (n = 217) of all medium metropolitan U.S. counties (Table 2). The number of CWSs ranged from 1,750 in large central metropolitan counties to 6,701 in medium metropolitan counties. For all contaminants combined, 2,071 (7%) CWS reported at least one annual mean greater than the MCL across all urbanization levels. The percentage of CWSs reporting at least one annual mean greater than the MCL (referred to as percentage of CWS greater than the MCL) generally increased from the most urban counties (5.4% in large central metropolitan counties) to the most rural (10% in noncore counties). Although small, the ORs for the probability a CWS reported at least one annual mean greater than the MCL were significant for medium metropolitan, small metropolitan, micropolitan, and noncore counties compared with large central metropolitan counties, adjusting for region, CWS size, and water source (Table 2). Significant ORs were found for medium metropolitan counties (OR: 1.004), small metropolitan counties (OR: 1.004), micropolitan counties (OR: 1.003), and noncore counties (OR: 1.005) compared with large central metropolitan counties.

**TABLE 2:** Water-quality measures in urban and rural counties — United States, 2010–2015

Contaminant*	County characteristics		Water-quality measure		
	Urban-rural category^†^	No. (%)^§^	No. of CWSs	CWS with one or more annual mean concentrations greater than MCL No. (%)	OR^¶^ (95% CI)
All	Large central metropolitan	39 (57)	1,750	94 (5.4)	Reference
	Large fringe metropolitan	173 (47)	5,557	314 (5.7)	1.002 (0.999–1.004)
	Medium metropolitan	217 (58)	6,701	493 (7.4)	**1.004 (1.002–1.006)**
	Small metropolitan	175 (48)	3,724	284 (7.6)	**1.004 (1.002–1.007)**
	Micropolitan	305 (47)	5,469	371 (6.8)	**1.003 (1.001–1.005)**
	Noncore	589 (43)	5,149	515 (10)	**1.005 (1.003–1.008)**
Arsenic (*µ*g/L)	Large central metropolitan	37 (54)	1,351	32 (2.4)	Reference
	Large fringe metropolitan	161 (43)	4,223	45 (1.1)	1.002 (0.990–1.015)
	Medium metropolitan	204 (54)	5,123	160 (3.1)	**1.015 (1.001–1.029)**
	Small metropolitan	163 (45)	2,703	65 (2.4)	1.011 (0.996–1.026)
	Micropolitan	272 (42)	3,936	66 (1.7)	1.005 (0.992–1.019)
	Noncore	542 (40)	3,738	77 (2.1)	1.010 (0.996–1.024)
Atrazine (*µ*g/L)	Large central metropolitan	36 (52)	1,126	0 (0)	—**
	Large fringe metropolitan	160 (43)	3,216	2 (0.06)	—
	Medium metropolitan	201 (53)	4,437	2 (0.05)	—
	Small metropolitan	162 (45)	2,361	0 (0)	—
	Micropolitan	268 (41)	3,470	1 (0.03)	—
	Noncore	528 (39)	3,263	1 (0.03)	—
DEHP (*µ*g/L)	Large central metropolitan	32 (47)	1,101	0 (0)	—
	Large fringe metropolitan	132 (35)	2,889	7 (0.2)	—
	Medium metropolitan	175 (46)	3,678	5 (0.1)	—
	Small metropolitan	136 (37)	1,900	2 (0.1)	—
	Micropolitan	227 (35)	2,686	3 (0.1)	—
	Noncore	382 (28)	2,264	5 (0.2)	—
HAA5 (*µ*g/L)	Large central metropolitan	31 (45)	1,055	10 (0.9)	Reference
	Large fringe metropolitan	164 (44)	4,168	39 (0.9)	1.002 (0.999–1.005)
	Medium metropolitan	204 (54)	4,513	84 (1.9)	**1.005 (1.002–1.009)**
	Small metropolitan	167 (46)	2,777	60 (2.2)	**1.006 (1.002–1.010)**
	Micropolitan	297 (46)	3,895	119 (3.1)	**1.011 (1.007–1.015)**
	Noncore	573 (42)	4,062	153 (3.8)	**1.013 (1.008–1.017)**
Nitrates (mg/L)	Large central metropolitan	36 (52)	1,494	8 (0.5)	Reference
	Large fringe metropolitan	156 (42)	4,633	8 (0.2)	0.999 (0.996–1.002)
	Medium metropolitan	191 (51)	5,606	69 (1.2)	1.003 (1.000–1.007)
	Small metropolitan	157 (43)	3,080	10 (0.3)	0.999 (0.996–1.002)
	Micropolitan	267 (41)	4,567	13 (0.3)	0.999 (0.996–1.002)
	Noncore	531 (39)	4,172	20 (0.5)	0.999 (0.996–1.002)
PCE (*µ*g/L)	Large central metropolitan	36 (52)	1,364	1 (0.07)	—
	Large fringe metropolitan	161 (43)	4,078	3 (0.07)	—
	Medium metropolitan	199 (53)	4,940	3 (0.06)	—
	Small metropolitan	163 (45)	2,780	2 (0.07)	—
	Micropolitan	273 (42)	4,064	0 (0)	—
	Noncore	535 (39)	3,867	3 (0.08)	—
Radium (pCi/L)	Large central metropolitan	27 (39)	638	11 (1.7)	Reference
	Large fringe metropolitan	119 (32)	2,171	68 (3.1)	1.003 (0.990–1.017)
	Medium metropolitan	153 (41)	2,109	45 (2.1)	0.993 (0.981–1.005)
	Small metropolitan	115 (32)	1,186	31 (2.6)	1.004 (0.986–1.022)
	Micropolitan	214 (33)	2,078	43 (2.1)	0.988 (0.974–1.002)
	Noncore	431 (32)	2,062	68 (3.3)	1.006 (0.989–1.023)
TCE (*µ*g/L)	Large central metropolitan	36 (52)	1,367	3 (0.22)	—
	Large fringe metropolitan	161 (43)	4,063	4 (0.1)	—
	Medium metropolitan	199 (53)	4,937	1 (0.02)	—
	Small metropolitan	163 (45)	2,776	1 (0.04)	—
	Micropolitan	272 (42)	4,062	0 (0)	—
	Noncore	534 (39)	3,866	1 (0.03)	—
TTHM (*µ*g/L)	Large central metropolitan	31 (45)	1,050	22 (2.1)	Reference
	Large fringe metropolitan	164 (44)	4,224	138 (3.3)	**1.006 (1.001–1.011)**
	Medium metropolitan	204 (54)	4,495	157 (3.5)	**1.009 (1.003–1.014)**
	Small metropolitan	167 (46)	2,759	127 (4.6)	**1.016 (1.009–1.022)**
	Micropolitan	297 (46)	3,889	162 (4.2)	**1.015 (1.009–1.021)**
	Noncore	572 (42)	4,128	271 (6.6)	**1.024 (1.017–1.032)**
Uranium (*µ*g/L)	Large central metropolitan	31 (45)	886	16 (1.8)	Reference
	Large fringe metropolitan	113 (30)	1,873	22 (1.2)	1.003 (0.986–1.021)
	Medium metropolitan	143 (38)	2,288	36 (1.6)	0.996 (0.979–1.013)
	Small metropolitan	87 (24)	891	26 (2.9)	1.027 (0.999–1.055)
	Micropolitan	169 (26)	1,538	23 (1.5)	1.003 (0.984–1.022)
	Noncore	241 (17)	1,032	22 (2.1)	1.003 (0.982–1.024)

**Abbreviations:** CI = confidence interval; CWS = community water system; DEHP = di(2-ethylhexyl) phthalate; GEE = generalized estimating equation; HAA5 = haloacetic acids; MCL = maximum contaminant level; MSA = metropolitan statistical area; OR = odds ratio; PCE = perchloroethene; TCE = trichloroethene; TTHM = total trihalomethanes.

* Data were obtained from the National Environmental Public Health Tracking Network and include data from 26 states during 2010–2015.

^†^ The metropolitan categories are large metropolitan (MSA population ≥1 million), medium metropolitan (MSA population 250,000–999,999) and small metropolitan (MSA population <250,000). Large metropolitan counties are subdivided into large central metropolitan counties and large fringe metropolitan counties based on the location and size of the MSA principal city. The nonmetropolitan categories are micropolitan (counties in a micropolitan statistical area defined as urban clusters with a population of 2,500–49,999) and noncore (counties not in a micropolitan statistical area) (**Source:** Ingram DD, Franco SJ. 2013 NCHS urban-rural classification scheme for counties. Vital Health Stat 2 2014;166:1–73).

^§^ Number represents the number of counties in data by urban-rural classification. Percent represents the percent of U.S. counties in that urban-rural classification in the data.

^¶^ ORs were calculated with GEE logistic regression and are adjusted for U.S. region, CWS size, and water source. Estimates in bold are significant (based on the 95% CIs).

** Values were not estimated because of a very low number of CWSs reporting one or more annual mean concentrations greater than the MCL.

Similar results were found for HAA5 and TTHM. For HAA5, the percentage of CWSs greater than the MCL increased from 0.9% in large central metropolitan counties to 3.8% in noncore counties. Significant ORs increased across urbanization level from medium metropolitan counties (OR: 1.005) to noncore counties (OR: 1.013). The percentage of CWSs greater than the MCL for TTHM increased from 2.1% in large central metropolitan counties to 6.6% in noncore counties but with small metropolitan counties slightly higher than micropolitan counties. Significant ORs were observed for large fringe metropolitan counties (OR: 1.006), medium metropolitan counties (OR: 1.009), small metropolitan counties (OR: 1.016), micropolitan counties (OR: 1.015), and noncore counties (OR: 1.024) compared with large central metropolitan counties.

The percentage of CWSs greater than the MCL for arsenic ranged from 1.1% in large fringe metropolitan counties to 3.1% in medium metropolitan counties. The OR for medium metropolitan counties compared with large central metropolitan counties was 1.015. No other ORs were significant for arsenic. For nitrates, the percentage of CWSs greater than the MCL ranged from 0.2% in large fringe metropolitan counties to 1.2% in medium metropolitan counties. For radium, the lowest percentage of CWSs greater than the MCL was in large central metropolitan counties (1.7%), and the highest was in noncore counties (3.3%) and large fringe metropolitan counties (3.1%). For uranium, the percentage ranged from 1.2% in large fringe metropolitan counties to 2.9% in small metropolitan counties. No significant ORs were found for nitrates, radium, and uranium. Logistic regression analysis results are not reported for atrazine, DEHP, PCE, and TCE because the number and percentage of CWSs reporting at least one annual mean greater than the MCL were very low, ranging from 0% to 0.2% of CWSs.

## Discussion

### Air

Ozone is a secondary pollutant generated by the reactions in sunlight of nitrogen oxides, volatile organic compounds, and carbon monoxide, which are largely emitted by the burning of fossil fuels (*15*). PM_2.5_ is either emitted directly into the atmosphere or forms from complex chemical reactions involving pollutants such as sulfur dioxides and nitrogen oxides (*16*). Sources of these air pollutants are typically more concentrated in urban areas, although pollutants can be carried downwind of urban sources and contribute to pollutant levels in surrounding areas. Air quality as measured by total number of ozone or PM_2.5_ days that are greater than the standard and average PM_2.5_ concentration improves significantly from the most urban to the most rural counties. Over 5 years, large central metropolitan counties experienced about 10 times the number of days greater than the ozone and PM_2.5_ standards than noncore counties. The differences between large central metropolitan counties and large fringe counties in the total number of days greater than the ozone or PM_2.5_ standard and average PM_2.5_ concentration also were significant. Across all levels of urbanization, the mean average annual PM_2.5_ concentration was below the annual PM_2.5_ standard of 12 *μ*g/m^3^ and improved from the most urban to the most rural counties. Although improvements have been made in air quality, the most urban counties still experience significantly worse air quality.

### Water

A total of 7.3% of the 28,350 CWSs represented reported one or more annual mean contaminant concentrations greater than the MCL for all 10 contaminants. The percentage of CWSs reporting one or more annual means greater than the MCL for all 10 contaminants generally increased from the most urban counties (5.4%) to the most rural counties (10.0%). Micropolitan counties, the second most rural category, had a lower percentage than both medium and small metropolitan counties. CWSs in noncore counties were twice as likely as CWSs in large central metropolitan counties to have one or more annual means greater than the MCL. Although this trend was significant after controlling for region, state, CWS size, and water source, the ORs were very small. For example, the odds of CWS having an annual mean greater than the MCL was only 0.5% higher in noncore counties than in large central metropolitan counties. Determining whether these differences affect public health is difficult, even though they were significant. The overall results might be driven largely by the trends observed for HAA5 and TTHM. Both pollutants are disinfection by-products formed when disinfectants added to drinking water react with naturally occurring substances such as organic matter in the source water (*18*,*22*). The increasing difference observed between large central metropolitan counties and less urban categories was observed after controlling for region, state, CWS size, and water source. CWSs in small metropolitan counties had a greater increase in annual means above the MCL for TTHM compared with large central counties than the less urban micropolitan counties. For both arsenic and nitrates, medium metropolitan counties had the highest percentage of CWSs reporting one or more annual means greater than the MCL. Arsenic is a naturally occurring element, whereas nitrates are released from nitrate-containing fertilizers, sewage and septic tanks, and decaying natural material such as animal waste. For arsenic, medium metropolitan counties had significantly higher ORs than urban counties even after controlling for region, state, CWS size, and water source. Radium is formed from the decay of uranium or thorium in the environment. Uranium is a naturally occurring element found in the Earth’s crust. No significant trends were found for uranium or radium after controlling for region, state, CWS size, and water source. The differences observed between rural and urban counties might be a result of differences in water treatment practices or related to financial challenges faced by rural CWSs, leading to workforce shortages, a lack of technical expertise, and an aging infrastructure (*21*,*23*).

## Limitations

These findings in this report are subject to several limitations. First, the results of such analyses can be influenced by how urbanization is defined and how counties are categorized, including the number of categories created. These results might differ from other analyses that are similar but have different categorization schemes. Second, counties are not homogeneous, and a county-based classification might not identify within-county variation. Third, although evaluating differences in air and drinking water quality based on EPA health standards can put those differences in the context of health, this method might overlook differences between counties in air or water contaminant concentration that are all greater than or all less than the standard. Fourth, an annual mean concentration might not identify differences in season, maximum, or cumulative concentrations. Fifth, county-level ozone and PM_2.5_ data might not adequately represent individual exposure resulting from pollutant heterogeneity and population movement within a county. Furthermore, the composition of PM_2.5_ can vary; therefore, data on the different components of PM_2.5_ (speciated data) might indicate additional differences by urbanization and by region. Sixth, the data available through the tracking network do not include data for all regulated contaminants in water, although the 10 available were selected because of their public health importance and frequency of detection and exceedance. Although the exceedances are typically low for the additional contaminants, including data for additional contaminants could change the results if exceedances for those contaminants vary strongly by urbanization. Lead was not included as one of the 10 contaminants because the potential difference in lead at the CWS compared with lead at the tap. Seventh, water samples from CWS are taken at entry points to the distribution system or representative sampling points after treatment. Depending on the contaminant, sampling results might not reflect concentration at the tap and might not reflect human exposure. Eighth, exposure to drinking water contaminants depends on tap water consumption and other personal behaviors that might vary by urbanization level. Ninth, these analyses adjust for spatial autocorrelation by including a fixed effect for state. More rigorous analyses that include enhanced modeling approaches might be needed to fully adjust for spatial autocorrelation in these data and to gain a more accurate estimate of the differences in air and water quality by urbanization. Finally, inferences based on these results are subject to potential multiple statistical testing errors.

## Future Directions

A more comprehensive analysis of these measures, with adjustments for spatial autocorrelation and an evaluation of regional and seasonal variation, might identify additional important differences between urban and rural counties in air and water quality. Additional enhancements and expansions in the nationally standardized environmental data would improve the ability to conduct such analyses to produce more comprehensive information. The tracking program will continue to work with federal, state, and local partners to improve the standardization of water-quality measures, including measures of concentration, and to expand air-quality measures to include speciated PM_2.5_ and source apportionment data.

## Conclusion

This report provides additional information about potential urban and rural differences in the physical aspects of social determinants of health by evaluating nationally standardized air- and water-quality data by a six-category urbanization scale. The findings indicate that air quality, measured by the total number of days greater than the ozone or PM_2.5_ standard and the mean PM_2.5_ concentration, improves as counties become more rural. Conversely, water quality measured by the occurrence of an annual mean greater than the MCL for 10 contaminants combined worsens as counties become more rural. Understanding these differences and their potential health impact can help to identify and prioritize potential environmental public health concerns and opportunities for action. Nationally standardized environmental health data can be used to identify vulnerable populations and areas of concern, develop policies, and focus interventions using a place-based approach.
